# Production and Biological Assessment of VNIISPK Cultivars of Various Ploidy for the Zone of Temperate Continental Climate

**DOI:** 10.3390/plants11202770

**Published:** 2022-10-19

**Authors:** Nina Krasova, Zoya Ozherelieva, Anna Galasheva, Maksim Lupin

**Affiliations:** Russian Research Institute of Fruit Crop Breeding (VNIISPK), 302530 Orel, Russia

**Keywords:** *Malus domestica*, adaptability, rootstock, apple scab, productivity, commercial qualities of fruits, taste

## Abstract

Increasing the reliability of fruit crops in unstable weather conditions of a temperate continental climate has become particularly relevant. This research was carried out based on a bioresource collection from the Russian Research Institute of Fruit Crop Breeding (VNIISPK BRC). Apple cultivars (diploids and triploids) of different maturation periods were studied. Triploid cultivars (3×) of summer maturation were developed using diploid gametes of the ‘Papirovka tetraploid’ cultivar as a donor (2-4-4-4×), triploids of winter maturation were developed using 13-6-106 (Suvorovetz: open pollination), and ‘Wealthy tetraploid’ (2-4-4-4×) or ‘Giant Spy’ (2-4-4-4×) were used as donors of diploid gametes. ‘Antonovka’ and ‘Melba’ were taken as control cultivars. The aim of this work was to evaluate the apple breeding cultivars according to the main economic and biological indicators and to determine the threshold of resistance to unfavorable conditions of the winter period for possible cultivation in specific conditions of a temperate continental climate. As a result of using the method of modeling damaging factors, apple cultivars that withstood not only the critical temperatures of January, but also especially dangerous frosts after thaws at the end of winter were identified, with the stability of vital tissues at the level of ‘Antonovka’ (a control cultivar), scab immune cultivars (RVi6) ‘Ivanovskoye’, ‘Zdorovie’ and ‘Pamyati Hitrovo’, and immune triploids (3×) ‘Vavilovskoye’, ‘Aleksandr Boyko’, Rozhdestvenskoye’ and ‘Academic Saveliev’. During the whole winter, the winter resistance of summer scab immune triploids, ‘Zhilinskoye’, ‘Maslovskoye’, ‘Yablochny Spas’ and ‘Spasskoye’, was at the level of ‘Antonovka’, but was higher than ‘Melba’. The high precocity of scab-immune cultivars, ’Afrodita’, ‘Ivanovskoye’, ‘Veniaminovskoye’, and ‘Yubiley Moskvy’, and triploids ‘Patriot’ and ‘Rozhdestvenskoye’, have been identified. Triploid cultivars are superior to diploid cultivars and control ‘Antonovka’ in fruit size. According to the complex of characteristics, promising apple cultivars were identified for planting in intensive orchards of the temperate continental climate zones, namely ‘Avgusta’ and ‘Solnyshko’ (summer cultivars), and ‘Aleksandr Boyko’, ‘Vavilovskoye’, ‘Venyaminovskoye’, ‘Ivanovskoye’ and ‘Rozhdestvenskoye’ (winter cultivars).

## 1. Introduction

Among fruit crops, apple has the greatest distribution and holds a leading place in fruit production in the temperate–continental climate zone. It has high adaptive abilities and is a valuable food product for humans. For successful gardening, it is necessary to select the right cultivars that meet these conditions and to ensure high profitability and high fruit quality.

Current intensification of horticulture is aimed at the dense placement of trees grown using low-growth clonal rootstocks, new cultivars and technologies that meet modern requirements and ensure the high profitability of production. The correct selection of cultivars that meet the modern requirements of production and consumers is of great importance in raising the profitability of apple plantations and increasing the quantity and quality of products [1,2,3,4,5,6,7].

In conditions of fierce competition and the appearance of high-quality foreign, but insufficiently winter-hardy, apple cultivars in the assortment of the middle gardening zone, the task is to attract new domestic cultivars that combine technology, precocity, high commodity and consumer qualities of fruits with high adaptability to unfavorable environmental factors [8,9,10,11,12].

Abiotic factors of the environment (early autumn–winter frosts, low critical temperatures in winter, prolonged frosts after thaws and solar heating with winter desiccation of fruit tree tissues, spring frosts, droughts and short vegetation period) cause great harm to apple trees and cause significant harm to agriculture. The impact of stress factors leads to a sharp decrease in the productivity and quality of fruits and even to the death of apple trees. The recent increase in cases of negative effects of various biotic and abiotic stress factors reinforce the role of new domestic apple cultivars that are adapted to certain growing conditions, with high resistance to the main fungal pathogens. For gardening in the temperate continental climate zone in Russia, the main limiting factor in the cultivation of high-quality apple cultivars are unfavorable factors for the autumn–winter period and lack of heat in summer. The effectiveness of gardening in a temperate climate zone largely depends on abiotic environmental factors. Increasing the reliability of fruit crops in unstable weather conditions of the temperate continental climate has become particularly relevant.

Periodically recurring severe winters cause significant damage to apple trees. Genetically determined winter hardiness depends on the natural conditions of the growing zone, on the combination of meteorological conditions in the autumn–winter period and on the degree of preparation of tissues in the autumn period. The hardy state of the tree develops in the process of hardening after the end of the shoot growth and entering dormancy under the influence of a gradually decreasing temperature and precipitation during this period [13,14,15].

A prerequisite for growing apple trees in a temperate continental climate zone is high frost resistance, which occurs during the hardening period in the autumn period. Stopping growth and going into a dormant state is an important condition for the adaptation of apple cultivars to unfavorable winter factors. In autumn, with a decrease in the activity of plant metabolism, under the influence of a gradual decrease in temperature and shortening of daylight, hardening occurs, the protective mechanisms of the wintering tree are activated, the energy potential to overwinter plants accumulates, the cytoplasm turns into a gel-like state, the water-holding capacity of tissues increases, and the amount of bound water also increases. The preparation of trees for winter is associated with the hydrolysis of starch, the accumulation of soluble sugars, proteins, and polyphenols in plant tissues, which increase the adaptive–protective process [16,17,18].

Winter hardiness of fruit plants is a complex property and consists of many components. In the middle zone of horticulture in Russia, winter damage to apple trees can occur in different periods; in early winter, with a possible drop in temperature to −25 °C, in mid-winter, with a drop in temperature to −40 °C, as well as frosts of −25 °C after a thaw of +2 °C at the end of winter.

The main factor in the adaptability of the cultivar is its productivity, with an increase in which other indicators of adaptation should also increase, i.e., winter hardiness, resistance to adverse conditions, diseases and pests, etc. Therefore, for high productivity and high quality of fruits not to depend on natural adverse factors, it is necessary to ensure a combination of all the necessary signs of adaptation at the maximum level in the cultivar [19,20].

One of the main indicators that characterize the value of the cultivar is the yield, which is determined by its biological and hereditary characteristics and largely depends on the growing conditions. The productivity potential (flower buds) of an apple tree begins at the end of the previous year; the formation of the yield goes through all the stages of organogenesis, from bud laying to fruit ripening. The realization of the productivity potential is closely related to abiotic and biotic environmental factors, which can significantly reduce the actual yield. The time of entry into fruiting and the rate of increase in the yield are very important.

The evaluation of quality indicators of apple cultivars is controlled by regulatory and technical documentation and methodology. Well-tested apple cultivars should be selected for each region, not only in terms of adaptability, but also in terms of fruit quality.

Intensive, low-growth apple tree plantings should be competitive in the domestic and global markets, and should ensure the high precociousness and productivity of cultivars with high fruit quality. The quality of fruits is currently subject to high requirements. Commodity and consumer qualities are determined by the pomological cultivar and growing conditions. The value of the cultivar is determined by its commercial qualities and the one-dimensionality of the fruits. Fruits that are attractive in appearance, with a beautiful cover color or golden yellow, medium or medium uniform size, with a smooth skin without rusting and a crisp dense juicy fragrant pulp and a rich sweet and sour–sweet taste are appreciated.

Intensive modern orchards need technological early-fruiting and high-yielding apple cultivars with a convenient growth habit. They must also be resistant to unfavorable abiotic and biotic factors, have high commercial and consumer qualities of fruits, be highly profitable, and provide a year-round supply of fresh fruits to the population of Russia.

Constantly changing requirements for cultivars and the arrival of new ones, which are the result of breeding and introduction, mean that there is a task to create a comprehensive, objective assessment of them in various regions.

The oldest pomological institution, VNIISPK (Orel), has collected a large gene pool of apples with various genetic and ecological–geographical origins, including local cultivars of folk selection, and new cultivars of domestic and foreign breeding. The Institute has created new domestic high-quality triploid and scab-immune cultivars (*RVi6*). According to numerous researchers, the number of modern innovative directions for apple breeding (the creation of cultivars that are scab-immune, columnar, etc.) includes the production of triploid cultivars [21,22,23,24]. These cultivars, which have different maturation periods, can provide a solution to this problem, but is subject to the careful study of adaptability, fertility, productivity, and commercial indicators of fruits in each specific zone.

This study allows us to evaluate the cultivars by winter hardiness and precocity to identify the best of them for modern intensive apple tree plantings in a specific zone of the temperate continental climate.

The goal of this work is to evaluate domestic apple cultivars of various origins according to the main production and biological indicators using a low-growing rootstock. We determined the threshold of resistance to adverse winter conditions. Isolated early-fruiting and disease-resistant cultivars, which combine high winter hardiness (at the level of the Central Russian cultivars) with high marketability, taste qualities and long shelf life (at the level of European cultivars) were evaluated.

## 2. Results

Cultivars planted in the spring of 2011 and 2012, in the apple variety plots studied, successfully endured the moderate winters of the first years of their growth in the garden.

In autumn, the studied cultivars underwent good hardening due to a gradual decrease in temperature (the average air temperature in September 2021 was 10.9 °C, in October it was 5.9 °C, in November it was 2.3 °C, and in December it was −4.6 °C), which contributed to the preparation of the studied cultivars for winter conditions.

To clarify the reaction of cultivars to possible low temperatures and to identify the threshold of resistance, artificial freezing of cultivars through winter hardiness components was carried out by modelling the damaging factors in order to determine their cultivation. As a result of artificial freezing, according to the components of frost resistance, good resistance to the early winter frosts of all the apple cultivars in the domestic selection was revealed, which, without damage, suffered a decrease in temperature after hardening (−5 °C, −10 °C) at the beginning of winter to −25 °C (Component I).

Buds, bark and wood at the beginning of winter were not damaged by frost −25 °C. When modeling the freezing temperature (−38 °C) in the middle of winter after quenching, the buds from the shoots were most vulnerable, with significant differences (*p* < 0.05) in the cultivars.

Bud damage was noted in the foreign cultivars, namely ‘Ligol’, ‘Melba’, and ‘Honeycrisp’, and domestic cultivars, namely ‘Start’ and ‘Patriot’ (2.4 points), more strongly than in ‘Antonovka’.

**Figure 1:**
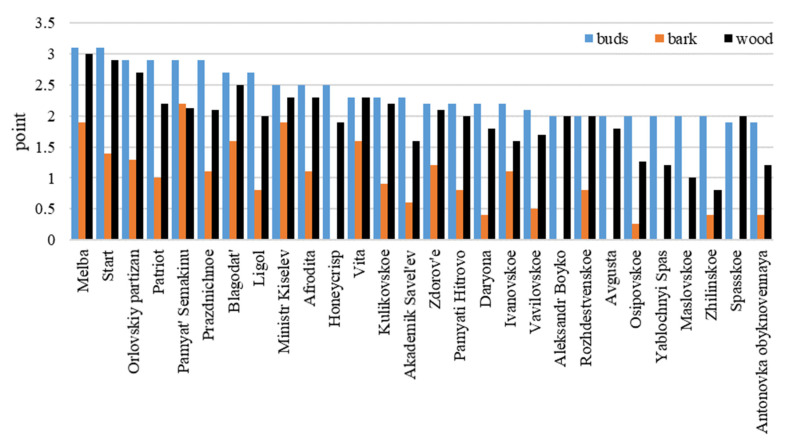
The degree of damage to the buds and tissues of shoots when modeling the frost at −40 °C (Component II. −5 °C, −10 °C, −40 °C) LSD05—0.3 (to buds); 0.4 (to bark and wood).

With the frost increasing to −40 °C, bud damage to these cultivars increased, especially to ‘Melba’ and ‘Start’—up to 3.0–3.1 points (Figure 1).

Significant damage to the wood was noted in ‘Melba’, ‘Blagodat’, Orlovsky Partizan’ and ‘Start’ (2.5–3.0 points).

Reversible damage to wood at −40 °C was detected in ‘Afrodita’ ‘Vita’ ‘Zdorovie’, ‘Kulikovskoye’ and triploids ‘Ministr Kiselev’ and ‘Patriot’ (2.1–2.4 points).

Weak damage to wood (at the level of ‘Antonovka’) was noted in ‘Academic Saveliev’, ‘Aleksandr Boyko’, ’Vavilovskoye’, ‘Ivanovskoye’, ‘Pamyat Hitrovo’, ‘Prazdnichnoye’, ‘Rozhdestvenskoye’, ‘Honeycrisp’ and in all the studied summer cultivars, except ‘Melba’. Bark damage in the studied cultivars was reversible, except for ‘Pamyat Semakinu’. Cambium showed good stability in most cultivars (lower than in Antonovka, but sufficient for the temperature of −40 °C).

Damage to an apple tree in winter can occur both as a result of critical frosts and frosts after thaws, as well as with sharp fluctuations in temperature during the day and at night, resulting in a decrease in the frost-resistant condition of the tree.

In February, modeling a thaw of +2 °C, followed by a decrease in temperature to −25 °C (Component III of frost resistance), revealed damage to buds and bark in most of the studied cultivars of VNIISPK breeding (Table 1). Winter cultivars ‘Academic Saveliev’, ‘Aleksandr Boyko’, ‘Afrodita’, ‘Blagodat’, ‘Ivanovskoye’, ‘Kulikovskoye’, ‘Prazdnichnoye’ and ‘Rozhdestvenskoye’ demonstrated high resistance of all the tissues at the level of ‘Antonovka’, while summer cultivars, ‘Daryona’ and ‘Yablochny Spas’, showed resistance at a higher level than ‘Melba’.

Buds and bark in ‘Vavilovskoye’, ‘Ministr Kiselev’, ‘Start’ and ‘Osipovskoye’ were damaged by frosts after the thaw to a lesser extent, but more strongly than in ‘Antonovka’.

Wood and cambium remained resistant to frost after thaws in all domestic cultivars, except for Start and Osipovskoye (weak damage).

The damage to buds and tissues in ‘Ligol’ and ‘Honeycrisp’ was significantly higher than in ‘Antonovka’ and most of the studied cultivars.

Thus, the resistance of buds and vital tissues of bark and wood with reversible damage was no stronger than that of ‘Antonovka’ when modeling the temperatures of −38 °C and −40 °C in mid-winter, and new scab-immune cultivars of winter maturation ‘Ivanovskoye’, ‘Zdorovie’, and ‘Pamyati Hitrovo’, as well as immune triploids ‘Aleksandr Boyko’, ‘Rozhdestvenskoye’, ‘Academic Saveliev’, ‘Vavilovskoye’ and ‘Prazdnichnoye’ demonstrated frost resistance to frosts of −25 °C after the simulated thaw of 2.0 °C at the end of winter.

Triploid cultivars of summer ripening, namely ‘Avgusta’, ‘Daryona’, and ‘Osipovskoye’, and immune triploids ‘Maslovskoye’ and ‘Zhilinskoye’. ‘Spasskoye’ and ‘Yablochny Spas’ withstood critical freezing temperatures in the middle of winter and frosts after thaws at the end of winter, with minor damage to vital tissues at the level of ‘Melba’. Hence, resistance to early winter temperature drops was identified in the cultivars of domestic breeding. In most, there were also temperature drops to −38 °C and −40 °C in the middle of winter, with reversible damage to bark, wood, cambium and they maintained a winter-hardy state to frost after thaws.

In ‘Ligol’ and ‘Honeycrisp’, bud damage in all the freezing modes was significantly stronger in ‘Antonovka’ and other studied cultivars with relative tissue stability.

An important indicator of a modern garden is the precocity of trees.

It was recorded that winter cultivars, namely ‘Afrodita’, ‘Blagodat’, ‘Ivanovskoye’, ‘Pamyati Hitrovo’, and ‘Rozhdestvenskoye’, and summer cultivars, namely ‘Zhilinskoye’, ‘Maslovskoye’, Solnyshko and ‘Yablochny Spas’ on semi-dwarf rootstock 54–118, started fruiting in the 3rd–4th year.

‘Afrodita’, ‘Blagodat’, Start’, ‘Ivanovskoye’, ‘Patriot’ and ‘Yubiley Moskvy’ at the age of five years yielded 13–18 kg per tree (8–10 t/ha). Six-year-old trees of ‘Afrodita’, ‘Zhilinskoye’, ‘Maslovskoye’ and ‘Spasskoye’ yielded 20–24 kg per tree (11–13 g/ha). Annual fruiting at this age was noted in ‘Venyaminovskoe’, ‘Rozhdestvenskoe’ and ‘Yubiley Moskvy’, with an increase in yield to 31–34 kg per tree (17–19 t/ha). In ‘Ivanovskoe’ and ‘Patriot’, at the age of 7 years, the yield was 17–20 kg per tree (see Table 2). Early entry into fruiting was also noted in ‘Rozhdestvenskoye’.

**Figure 2:**
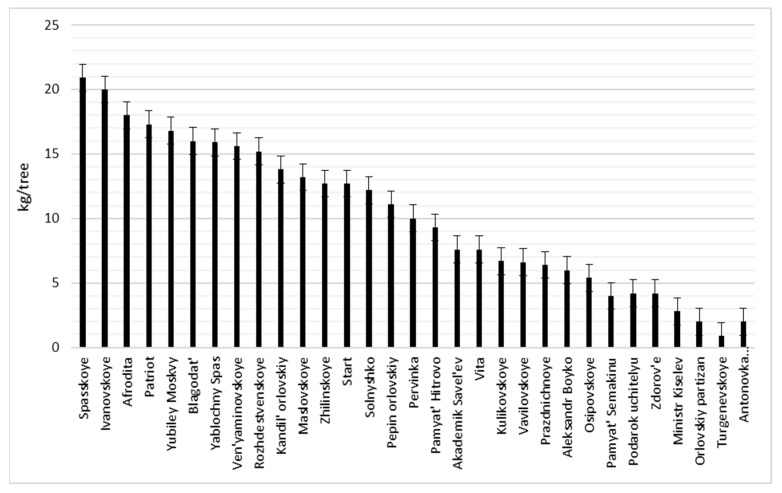
Average yield of the cultivars for 2019–2021 (LSD_05_ = 10.4).

On average, during the study period, triploids ‘Blagodat’, ‘Patriot’, scab-immune cultivars ‘Afrodita’, ‘Venyaminovskoye’, ‘Ivanovskoye’, Solnyshko,‘Start’, scab-immune triploids ‘Rozhdestvenskoye’, ‘Zhilinskoye’, ‘Candil Orlovsky’, ‘Start’, ‘Maslovskoye’,’ Spasskoye’ and ‘Yablochny Spas’ significantly surpassed the productivity of the rest of the studied cultivars, the yield of which was at the level of ‘Antonovka’ (Figure 2).

Late entry into fruiting was noted in ‘Zdorovie’, ‘Ministr Kiselev’, ‘Orlovsky Partizan’, ‘Pamyat Semakinu’, ‘Podarok Uchitelyu’ and ‘Turgenevskoye’.

Below is a brief description of the apple cultivars studied on the semi-dwarf rootstock 54–118 (Table 2).

The evaluation of quality indicators of apple cultivars is controlled by regulatory and technical documentation and methodology. Well-tested apple cultivars should be selected for each region, not only in terms of adaptability, but also in terms of fruit quality.

Intensive, low-growth apple tree plantings should be competitive in the domestic and global markets and ensure the high precociousness and productivity of cultivars with high fruit quality. The quality of fruits is currently subject to high requirements. Commodity and consumer qualities are determined by the pomological cultivar and growing conditions. The value of the cultivar is determined by its commercial qualities and the one-dimensionality of the fruits. Fruits that are attractive in appearance, with a beautiful cover color or golden yellow, medium or medium uniform in size, with a smooth skin without rusting and a crisp dense juicy fragrant pulp and a rich sweet and sour–sweet taste are appreciated.

The study of apple cultivars, according to the commercial and consumer qualities of fruits, allowed us to identify the largest fruits in triploid cultivars: ‘Aleksandr Boyko’, ‘Rozhdestvenskoye’ and ‘Yablochny Spas’ (with an average fruit weight of 205–210 g). Triploids, namely ‘Academic Saveliev’, ‘Blagodat’, ‘Vavilovskoye’, ‘Ministr Kisilev’, ‘Orlovsky Partizan’, ‘Pamyat Semakinu’, ‘Patriot’ and ‘Prazdnichnoye’, have fruit of above the average weight. Summer cultivars, namely ‘Zhilinskoye’, ‘Maslovskoye’ and ‘Spasskoye’, also have fruit of above the average weight (155–170 g).

**Figure 3:**
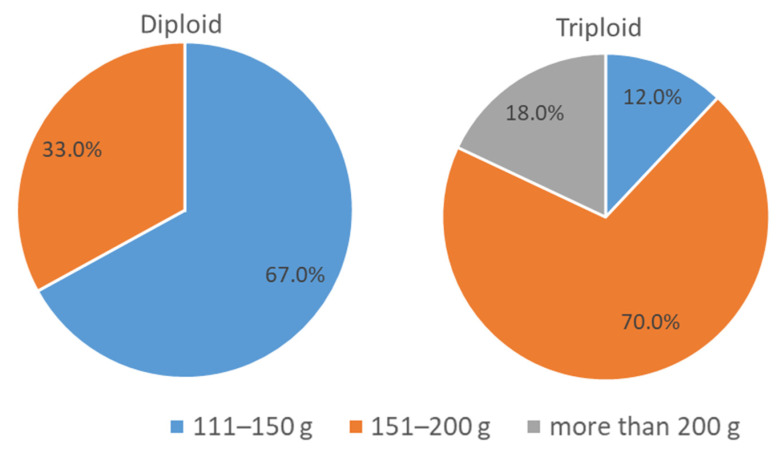
Division of diploid and triploid cultivars by average fruit weight (%).

In the triploid group, cultivars with large fruits, with an average weight of 151–210 g and above average weight, prevail; in the diploid group, most cultivars have an average fruit weight of 140–150 g (Figure 3) and only ‘Veniaminovskoye’, ‘Vita’, ‘Ivanovskoye’, ‘Pamyati Hitrovo’ and ‘Solnyshko’ have a fruit weight above average size. The differences in fruit weight between the groups of triploids and diploids are significant are in accordance with the calculation of the U-test criterion (Mann-Whitney, U-test) [25].

Consumers primarily evaluate apples by their appearance, absence of damage by diseases and pests, one-dimensionality, size. shape, color, and taste, and consumers prefer their familiar and favorite cultivars.

On the shop shelves of Russia, most of the new breeding cultivars are not inferior to imported fruits in terms of marketable qualities and the attractiveness of fruits. The fruits ‘Ivanovskoe’ and ‘Pamyat Semakinu’ are very attractive in appearance, with a bright red solid blush. ‘Aleksandr Boyko’, ‘Afrodita’, ‘Veniaminovskoe’ and ‘Ministr Kiselev’ have an intense and bright crimson–red blush on the entire surface of the fruit.

‘Rozhdestvenskoye ‘and ‘Prazdnichnoye’ have very beautiful fruits, with a thick dark red blush and a burgundy shade. ‘Vavilovskoye’ has large marketable fruits with a blurry-striped red blush and ‘Blagodat’ has a crimson–red blush.

With a long shelf life of fruits (until March), ‘Aleksandr Boyko’ stands out. The fruits of ‘Vavilovskoye’ and ‘Prazdnichnoe’ are stored until February, the fruits of ‘Ivanovskoe’, ‘Pamyat Semakinu’, ‘Venyaminovskoe’ and ‘Ministr Kiselev have a shelf life until the end of January, preserving the freshness and juiciness of the pulp. According to the taste qualities, these cultivars are rated as a dessert by 4.5 points.

Summer cultivars, namely ’Osipovskoye’, ‘Spasskoye’ Avgusta’ and ‘Solnyshko’, have very attractive fruits, with a bright crimson–red blush, great taste and juicy fragrant pulp.

## 3. Discussion

The negative effects of various biotic and abiotic environmental stress factors significantly reduce the size and quality of fruit yield and cause great damage to fruit plants.

Therefore, the problem of creating and selecting new, high-quality domestic apple cultivars for industrial cultivation with high adaptability in unstable conditions is particularly relevant [19]. In the horticulture industry, this problem is particularly acute, with a large pesticide load associated with a large number of protective measures in orchards. This contributes to a decrease in the ecological purity of the resulting products. In changing conditions, adaptive apple cultivars, which are resistant to stress factors, major diseases, stable fruiting, and high-quality fruits are needed. Previously popular apple cultivars, namely ‘Orlik’, ‘Orlovskoye Polosatoye’, ‘Veteran’, ‘Pepin Orlovsky’, ‘Pervinka’, ‘Pamyat Voinu’ and others, have lost their significance due to the size reduction of fruits and increased damage by diseases and pests. The widespread use of high-quality Western European cultivars in industrial plantings is impossible due to their low resistance to the harsh cold of the autumn–winter period, to the spring frosts during flowering and the lack of heat in summer in the central zone of Russia, which is consistent with the data obtained by a number of authors [26,27]. The creation of scab-immune apple cultivars and the use of polyploidy, with heterochromosomal crosses, offers great prospects for the creation of new genotypes with valuable production and biological indicators. Russian Research Institute of Fruit Crop Breeding has created scab-immune cultivars and triploid apple cultivars. Of particular value are triploid cultivars that have immunity to the most common apple scab diseases (*RVi6*). Therefore, a comprehensive approach is needed to evaluate new and innovative apple cultivars to determine the potential for resistance and commercial qualities of fruits in modern conditions on a certain rootstock.

The results of artificial freezing allowed us to determine high resistance to early winter frosts in all the studied domestic cultivars. In the middle of winter, when the frost was −40 °C, the buds and wood were more damaged, while the bark was more stable for most cultivars since it lost stability more slowly. During the thaw at the end of winter, the vegetative buds and bark of most cultivars lost stability and were more damaged by frost. ’Ivanovskoye‘, ‘Zdorovie’, ’Pamyati Hitrovo‘ (scab immune diploids of winter maturation), ‘Aleksandr Boyko’, ‘Rozhdestvenskoe’, ‘Academic Saveliev’, ‘Vavilovskoe’ and ‘Prazdnichnoye’ (scab immune triploids), as well as summer triploid ‘Avgusta’, demonstrated frost resistance of vegetative buds and tissues at the level of ‘Antonovka’, according to all the components of winter hardiness. During winter, the resistance of summer scab immune triploids, namely ‘Zhilinskoye’, ‘Maslovskoye’, ‘Yablochny Spas’ and ‘Spasskoye’, was at the level of ‘Antonovka’, but above the level of ‘Melba’. The analysis of variance showed significant differences between the apple cultivars.

Thus, the frost resistance of domestic cultivars to early winter temperature drops was identified. Most of them were found to be resistant to temperature drops to −38 °C and −40 ° C in the middle of winter with reversible damage to bark, wood and cambium, and they also maintained a winter-hardy condition to frost after thaws.

High precocity of scab-immune ‘Afrodita’, ‘Ivanovskoye’,’ Venyaminovskoye’, and ‘Yubiley Moskvy’, and triploids ‘Patriot’ and ‘Rozhdestvenskoye’, has been proven.

According to E.N. Sedov et al., “Triploid cultivars are characterized by high productivity, high commercial and consumer qualities of fruits, more regular fruiting, increased self-fertility, and in winter hardiness and resistance to diseases are not inferior to diploid cultivars” [28]. According to the data obtained, the studied group of triploids significantly exceeded diploids in terms of fruit weight, and were not inferior to imported fruits in terms of attractiveness and beautiful cover color.

In general, triploids surpass diploids and the control cultivar ‘Antonovka’ in the size of fruits.

A number of authors have reported on the significant influence of growing conditions and agronomic factors on the quality of fruits (fruit size, pulp density, and soluble solids content). [29,30,31]. The fruits of foreign apple cultivars, ‘Honeycrisp’ and ‘Ligol’, which are grown in the orchards of the central zone of Russia, have a dense juicy pulp and are stored for a long period, but the intensity of taste due to the lack of sunny days and heat during the ripening period is significantly inferior to the cultivars of local breeding.

## 4. Methods and Materials

This study was carried out in 2012–2021 at the site of the apple variety study by VNIISPK. The Orel region is located in a temperate continental climate zone in the central part of the Central Russian Upland at an altitude of 203 m above sea level.

The average annual long-term air temperature is 4.6 °C, the average temperature of winter months is −8.4 °C, and the absolute minimum air temperature in winter is −39.9 °C. The absolute maximum in summer is +40 °C, the sum of temperatures above +10 °C is 2250 °C, and the growing season lasts 175–185 days. The average annual precipitation is 550 mm, and droughts and dry winds are common in spring.

The analysis of meteorological data over the past 20 years has shown unstable weather conditions in the temperate continental climate of the Orel region. According to long-term data, the average temperature of the winter months was −8.4 °C, but this indicator was very unstable over the years. For the period from 2002 to 2022, there were significant fluctuations in this indicator, from −10.2 °C (winter 2002/2003) to −0.4 °C (Table 3) (winter 2019/2020) (data from the VNIISPK weather station). Cold winter periods were replaced by moderately warm ones. Numerous researchers reported on the impact of climate change on fruit plants [32].

Warm winters have become more frequent in recent years, and our research coincided with the period of warm winters, when the sum of negative winter temperatures was very small (for example, in the winter of 2019/2020, it was only 112.6 °C), and the minimum temperature did not fall below −15 °C. Therefore, it was necessary to carry out artificial freezing on the components of winter hardiness to establish a threshold for the frost resistance of new cultivars and to clarify the possibilities of their use in intensive plantings.

The object of this study was 32 apple cultivars (diploids and triploids) of different maturation periods (summer, autumn, and winter) and control cultivars ‘Melba’ and ‘Antonovka’.

This study included triploid cultivars of summer ripening, namely ‘Avgusta’, ‘Daryona’, ‘Maslovskoye’, ‘Osipovskoye’ and ‘Yablochny Spas’, which developed by using diploid gametes of ‘Papirovka tetraploid’ as a donor (2-4-4-4×). Triploids of winter maturation, ‘Orlovsky Partizan’ and ‘Patriot’, were obtained using a donor of diploid gametes 13-6-106 (Suvorovets—open pollination). Using ‘Wealthy tetraploid’ (2-4-4-4×), triploid ‘Ministr Kiselev’ and two immune cultivars (*RVi6*), ‘Aleksandr Boyko’ and ‘Vavilovskoe’, were developed. ‘Blagodat’ and ‘Prazdnichnoye’ were created using ‘Giant Spy’.

The triploids, namely ‘Pamyat Semakiny, ‘Sinap Orlovsky’, ‘Rozhdestvenskoye’ and ‘Yubilar’, were obtained by crossing diploid cultivars, one of which was prone to the formation of non-reduced gametes [24].

This experiment was undertaken in the autumn of 2014. The planting scheme was 6 × 3 m. The rootstock was a semi-dwarf clone 54–118.

Generally accepted methods of variety study in the field and laboratory conditions were used [33,34].

To determine the differences between the groups of diploids and triploids, the nonparametric Mann-Whitney U-test method was used [25].

U exp = n_1_ · n_2_ + [n_x_ · (n_x_ + 1)]/2 − T_x_

n_1_—the number of elements of the first sample

n_2_—the number of elements of the second sample

T_x_—greater of the two rank sums

Winter hardiness was studied in the laboratory conditions in a five-fold repetition by modeling damaging factors in the environmental test chamber ESPEC PSL-2KPH.

Damage to bark, wood and cambium was assessed by the degree of tissue browning on the longitudinal section. The intensity of browning of the tissues corresponded to the degree of freezing. Undamaged tissue had a light green color and damage caused darkening of tissues to varying degrees (from light yellow to dark brown or black). Tissue damage on frozen branches was assessed on a five-point scale by the degree of darkening, where 0 was the absence of damage, 1 was minor damage with a slight darkening up to 10–15% of the tissue area, 2 was reversible damage with a darkening up to 40% of the tissue area, 3 was up to 60% of the area, 4 was severe damage with a darkening of 60–80% of the area, which was not capable of tissue regeneration, and 5 was more than 80% of the area had a dark brown color and was dead.

The assessment of the degree of bud damage was assessed on a scale: where 0 was no damage; 1 was insignificant damage, and the tissue under the bud was damaged; 2 was reversible damage, and a part of the leaf bud was damaged; 3 was moderate damage, and the vascular system and most of the leaf buds were damaged; 4 was severe damage, and apical meristems and most of leaf buds were dead; 5 was buds and tissues were dead.

The simulation of freezing conditions was carried out after gradual hardening at a temperature of −5 °C and −10 °C (5 days) under the following modes of the main components of frost resistance:

I—resistance to early frosts in early December, −5 °C, −10 °C, and −30 °C;

II—the maximum level of frost resistance in January –February (freezing mode: −5 °C, −10 °C, −38 °C and −5 °C, −10 °C, and −40 °C);

III—resistance to frosts after thaw (freezing mode: −5 °C, −10 °C, +2 °C, and −25 °C).

Temperature decrease by 5 °C per hour; critical temperatures were maintained for 8 h; thaw (+2 °C) was 2 days.

To group fruits by size, the following gradation was followed: medium—111–150 g; above average—151–200 g; large—201–250 g; and very large—251–350 g [35].

The appearance of fruits and taste were evaluated at the tastings during the period of optimal maturity.

The tasting commission consisted of seven highly qualified employees, the assessment was given individually, and then the average score was calculated.

The taste qualities of the fruits were determined by taking into account the following scale: dessert taste—4.5 points and above, excellent table taste, 4.3–4.4 points, good taste, 4.2–4.1 points, 4.0 and below, mediocre taste.

The cultivars were evaluated by one-dimensionality, juiciness, appearance of attractiveness and duration of the consumer period. According to these indicators, the cultivars were compared with the control ones, with each other and with imported cultivars during the optimal maturation period.

Statistical data processing was carried out by generally accepted methods using the Microsoft Office Excel program [36,37].

## 5. Conclusions

According to the complex of characteristics, promising apple cultivars were identified for planting in intensive orchards of the temperate continental climate zone: ‘Avgusta’ and ‘Solnyshko’ (summer cultivars); ‘Aleksandr Boyko’, ‘Vavilovskoye’, ‘Venyaminovskoye’, ‘Ivanovskoye’ and ‘Rozhdestvenskoye’ (winter cultivars).

The cultivation of new, adaptive domestic apple cultivars with a complex of basic production and biological characteristics and high quality of fruits will contribute to improving environmental safety and the economic efficiency of horticulture.

## Figures and Tables

**Table 1:** The degree of damage to apple cultivars when modeling −25 °C frost after +2 °C thaw (Component III of winter hardiness).

Cultivar	−5 °C, −10 °C, +2 °C, −25 °C, point			
	Buds	Bark	Wood	Cambium
Winter cultivars				
Academic Saveliev	0.6	0.0	0.0	0.0
Aleksandr Boyko	0.8	0.4	0.0	0.0
Antonovka	0.4	0.0	0.0	0.0
Afrodita	1.0	0.0	0.0	0.0
Blagodat	0.2	0.0	0.0	0.0
Vavilovskoye	1.5	0.7	0.0	0.0
Vita	0.6	0.5	0.0	0.0
Zdorovie	0.8	0.4	0.0	0.0
Ivanovskoye	0.4	0.0	0.0	0.0
Kulikovskoye	0.2	0.0	0.0	0.0
Ligol	2.0	1.0	0.4	1.0
Ministr Kiselev	1.2	0.4	0.0	0.0
Orlovsky Partizan	1.0	0.2	0.0	0.0
Pamyati Hitrovo	1.4	1.0	0.0	0.0
Pamyat Semakinu	1.0	0.6	0.0	0.0
Patriot	0.6	0.2	0.0	0.0
Prazdnichnoye	0.4	0.0	0.0	0.0
Rozhdestvenskoye	0.4	0.4	0.0	0.0
Start	1.3	1.0	0.4	0.0
Honeycrisp	2.2	1.3	0.0	0.6
Summer cultivars				
Avgusta	0.5	0.1	0.0	0.0
Daryona	0.6	0.0	0.0	0.0
Zhilinskoye	1.0	0.5	0.0	0.0
Maslovskoye	0.7	0.3	0.0	0.0
Melba	1.5	0.3	0.3	0.0
Osipovskoye	1.8	1.1	0.4	0.6
Spasskoye	0.6	0.2	0.0	0.0
Yablochny Spas	0.6	0.0	0.0	0.0
LSD _05_	0.61	0.45	-	-

**Table 2:** Evaluation of apple cultivars by the main economic and biological indicators (2018–2021). Planting in autumn 2014.

Cultivar	Average Yield, kg per Tree	Fruit Ripening Period	Resistance to Scab	Fruit Taste, Point	Fruit Juiciness	Average Fruit Weight, g	Fruit Storage Duration
Triploids							
Avgusta (3×)	4.1	late summer	middle	sourish-sweet/4.4	juicy	160	20–25 days
Academic Saveliev (3×)	7.5	winter	high (*RVi6*)	sour-sweet/4.3	juicy	160	late January
Aleksandr Boyko (3×)	6.0	late winter	high (*RVi6*)	dessert/4.5	juicy	210	till early March
Blagodat (3×)	16.0	early winter	middle	dessert, sour-sweet/4.4	very juicy	170	late December
Vavilovskoye (3×)	6.6	winter	high (*RVi6*)	dessert/4.5	very juicy	180	till late February
Zhilinskoye (3×)	12.7	summer	high (*RVi6*)	dessert, sour-sweet/4.4	juicy	160	15–20 days
Maslovskoye (3×)	13.2	summer	high (*RVi6)*	sour-sweet/4.2	middle juicy	155	20 days
Ministr Kiselev (3×)	2.8	winter	resistant	sour-sweet/4.5	juicy	170	late January
Osipovskoye (3×)	5.4	early summer	middle resistant	sour-sweet/4.4	juicy	140	10–15 days
Orlovskiy partizan(3×)	2.0	winter	resistant	sour-sweet/4.4	juicy	165	late January
Pamyat Semakinu (3×)	4.2	early winter	resistant	sour-sweet/4.2	juicy	160	till January
Patriot (3×)	17.3	winter	middle	sour-sweet/4.4	juicy	180	till early February
Prazdnichnoye (3×)	6.4	early winter	high (*RVi6*)	dessert/4.4	juicy	180	till December
Rozhdestvenskoye (3×)	15.2	winter	high (*RVi6*)	dessert sour-sweet/4.5	very juicy	210	late January
Spasskoye (3×)	20.9	summer	high (*RVi6*)	sour-sweet/4.3	juicy	155	10–15 days
Turgenevskoye (3×)	0.9	winter	middle	sour-sweet/4.3	juicy	150	till January
Yablochny Spas (3×)	15.9	summer	high (*RVi6*)	sour-sweet/4.4	very juicy	205	15–20 days
Diploids							
Antonovka	2.0	early winter	middle	sweey-sour/4.2	juicy	150	late November
Afrodita	18.0	early winter	high *(RVi6*)	sour-sweet/4.4	juicy	150	late December
Venyaminovskoye	15.6	winter	high (*RVi6*)	sour-sweet/4.3	juicy	155	late February
Vita	7.6	winter	high field resistance	sour-sweet/4.2	juicy	160	till early February
Zdorovye	4.0	winter	high (*RVi6*)	sour-sweet/4.3	middle juicy	140	till February
Ivanovskoye	20.0	winter	high (*RVi6*)	sour-sweet/4.4	juicy	165	late January
Candil Orlovskiy	13.8	early winter	high (*RVi6*)	sour-sweet/4.4	middle juicy	140	till January
Kulikovskoye	6.7	late winter	high field resistance	sourish-sweet/4.3	middle juicy	140	till April
Pamyati Hitrovo	9.3	winter	high *(RVi6*)	sour-sweet/4.3	middle juicy	170	till February
Pepin Orlovskiy	11.1	early winter	high field resistance	sour-sweet/4.4	juicy	150	till January
Pervinka	10.0	autumn	high	sour-sweet/4.3	very juicy	140	till early November
Podarok Uchitelyu	4.2	summer	middle	sour-sweet/4.4		140	10–15 days
Solnyshko	12.2	late summer	high (*RVi6*)	sour-sweet/4.3	juicy	160	till November
Start	12.7	winter	high (*RVi6*)	sour-sweet/4.3	juicy	140	mid-January
Yubiley Moskvy	16.8	early winter	high (*RVi6*)	sour-sweet/4.3	juicy	140	late December
LSD _05_	10.4						

Note: (3×)—triploid.

**Table 3:** Winter conditions in 2002–2022 (data of VNIISPK weather station).

Years	Average Temperature of Winter Months (Average Annual −8.4 °C)	Sum of Average Daily Negative Temperatures, °C	Minimal Air Temperature, °C	Number of Days with Thaws
2002/2003	−10.2	1099.6	−30.5	8
2003/2004	−5.2	502.5	−24.5	17
2004/2005	−4.9	746.8	−26.5	21
2005/2006	−9.3	1198.1	−36,5	6
2006/2007	−3.1	421.0	−27.2	43
2007/2008	−4.2	424.4	−21.2	15
2008/2009	−4.9	536.4	−19.5	16
2009/2010	−9.8	1033.9	−32.0	23
2010/1011	−8.6	946.0	−34.2	5
2011/2012	−6.8	694.1	−39.9	26
2012/2013	−6.6	951.5	−31.7	35
2013/2014	−5.9	576.1	−31.0	15
2014/2015	−5.1	486.5	−24.5	9
2015/2016	−4.3	499.3	−29.3	18
2016/2017	−6.6	601.3	−24.4	5
2017/2018	−4.9	695.0	−26.1	18
2018/2019	−5.0	554.6	−24.5	4
2019/2020	−0.4	112.6	−15.0	21
2020/2021	−6.7	669.5	−29.3	9
2021/2022	−5.4	371.7	−20.1	6

## Data Availability

The data presented in this study are available upon request from the corresponding author.
